# Nanodrug-Enhanced Radiofrequency Tumor Ablation: Effect of Micellar or Liposomal Carrier on Drug Delivery and Treatment Efficacy

**DOI:** 10.1371/journal.pone.0102727

**Published:** 2014-08-18

**Authors:** Marwan Moussa, S. Nahum Goldberg, Gaurav Kumar, Rupa R. Sawant, Tatyana Levchenko, Vladimir P. Torchilin, Muneeb Ahmed

**Affiliations:** 1 Laboratory for Minimally Invasive Tumor Therapies, Department of Radiology, Beth Israel Deaconess Medical Center/Harvard Medical School, Boston, MA, United States of America; 2 Division of Image-guided Therapy and Interventional Oncology, Department of Radiology, Hadassah Hebrew University Medical Center, Jerusalem, Israel; 3 Department of Pharmaceutical Sciences and Center for Pharmaceutical Biotechnology and Nanomedicine, Northeastern University, Boston, MA, United States of America; Faculté de médecine de Nantes, France

## Abstract

**Purpose:**

To determine the effect of different drug-loaded nanocarriers (micelles and liposomes) on delivery and treatment efficacy for radiofrequency ablation (RFA) combined with nanodrugs.

**Materials/Methods:**

Fischer 344 rats were used (n = 196). First, single subcutaneous R3230 tumors or normal liver underwent RFA followed by immediate administration of IV fluorescent beads (20, 100, and 500 nm), with fluorescent intensity measured at 4–24 hr. Next, to study carrier type on drug efficiency, RFA was combined with micellar (20 nm) or liposomal (100 nm) preparations of doxorubicin (Dox; targeting HIF-1α) or quercetin (Qu; targeting HSP70). Animals received RFA alone, RFA with Lipo-Dox or Mic-Dox (1 mg IV, 15 min post-RFA), and RFA with Lipo-Qu or Mic-Qu given 24 hr pre- or 15 min post-RFA (0.3 mg IV). Tumor coagulation and HIF-1α orHSP70 expression were assessed 24 hr post-RFA. Third, the effect of RFA combined with IV Lipo-Dox, Mic-Dox, Lipo-Qu, or Mic-Qu (15 min post-RFA) compared to RFA alone on tumor growth and animal endpoint survival was evaluated. Finally, drug uptake was compared between RFA/Lipo-Dox and RFA/Mic-Dox at 4–72 hr.

**Results:**

Smaller 20 nm beads had greater deposition and deeper tissue penetration in both tumor (100 nm/500 nm) and liver (100 nm) (p<0.05). Mic-Dox and Mic-Qu suppressed periablational HIF-1α or HSP70 rim thickness more than liposomal preparations (p<0.05). RFA/Mic-Dox had greater early (4 hr) intratumoral doxorubicin, but RFA/Lipo-Dox had progressively higher intratumoral doxorubicin at 24–72 hr post-RFA (p<0.04). No difference in tumor growth and survival was seen between RFA/Lipo-Qu and RFA/Mic-Qu. Yet, RFA/Lipo-Dox led to greater animal endpoint survival compared to RFA/Mic-Dox (p<0.03).

**Conclusion:**

With RF ablation, smaller particle micelles have superior penetration and more effective local molecular modulation. However, larger long-circulating liposomal carriers can result in greater intratumoral drug accumulation over time and reduced tumor growth. Accordingly, different carriers provide specific advantages, which should be considered when formulating optimal combination therapies.

## Introduction

Radiofrequency ablation (RFA) is now a mainstay treatment for primary and secondary small focal tumors in the liver, lung, kidney, and other organs, with long-term studies demonstrating good outcomes in well-selected patient populations [1], [2]. However, challenges to RF ablation of larger tumors remain, including the potential persistence of residual tumor cells within the ablation zone and the surrounding ablative margin despite apparent adequate treatment [3], [4]. Therefore, strategies to target residual viable tumor cells and achieve a more complete treatment are being actively pursued. One such strategy has been to combine RF ablation with chemotherapy delivered in liposomal nanocarriers to target partially-injured viable cells in the ablation zone and surrounding periablational rim [5]–[7]. Early studies demonstrate increased local tumor coagulation, intratumoral drug accumulation, increased animal endpoint survival, and increased tumor coagulation in clinical studies using long-circulating liposomal doxorubicin as an adjuvant to RF [5]–[9].

Over the last several years, greater mechanistic understanding of the RFA-induced tissue reactions and low-level hyperthermia in the periablational margin has led to refined approaches such as modulating chemotherapy drug payload, composition, and liposomal drug release profile [10]–[15]. Key examples include the use of liposomal quercetin to eliminate upregulated heat shock proteins and bortezomib to eliminate HIF-1a and thereby increase tumor destruction [14], [16]. Yet, limitations persist, either in the form of incomplete modulation of target post-RFA tissue reactions, inadequate spatial and temporal coordination of drug delivery to the periablational rim, or sub-optimal drug release [14], [17], [18]. Specifically, in the case of liposomal quercitin, although marked reduction in the thickness of the rim of HSP was noted, persistence of more peripheral expression of HSP was seen. This provides ample rationale for further study to uncover the optimal nanocarriers to be used in the setting of ablation.

Most studies have used 100 nm-size liposomal carriers, based upon original combination therapy studies and the long-circulating nature of many of these formulations [12], [18]. Yet, within the fields of oncology and pharmacotherapeutics, there is increasing interest in using smaller carriers (micelles and spheroids) to improve intratumoral drug delivery and deeper interstitial penetration on its own, and in combination with low-level generalized hyperthermic treatments [19], [20]. However, such carrier alterations will likely affect properties of drug delivery such as kinetics, warranting formal evaluation of potential trade-offs between different outcomes (e.g., delivery, modulation of specified targets, and overall survival).

Along these lines, here, we sought to determine whether or not we could alter the nanodrug formulation to improve the spatial distribution of specific drugs to target relatively “short-acting” processes in a rim further from the ablation zone that were inadequately treated using long-circulating liposomes in prior studies [14], [21]. Accordingly, we studied the comparative effects of smaller (20 nm beads or micelles) and larger–sized (100+ nm beads or long-circulating PEG-coated liposomes) particles/carriers: on 1) distribution in the periablational rim using fluorescent beads (to determine the extent to which smaller particles have deeper penetration in periablational inflammatory tissue; 2) suppressing key ablation-induced reactions including pro-angiogenic hypoxia-inducible factor (HIF-1α) and protective heat shock protein (HSP70) production in the periablational rim using targeted drug payloads (doxorubicin/Dox and quercetin/Qu); 3) intratumoral drug accumulation of a target drug payload (doxorubicin); and finally 4) determine whether any of these primary end-points ultimately affected tumor growth rate and animal endpoint survival.

## Materials and Methods

### Experimental Overview

All animal work was conducted according to relevant national and international guidelines. Approval of the Beth Israel Deaconess Medical Center Institutional Animal Care and Use Committee was obtained prior to the start of this study. The study was performed in four phases. A total of 196 female Fischer 344 rats were used. All drugs were administered intravenously (IV). The following abbreviations are used: liposomal doxorubicin (Lipo-Dox), micellar doxorubicin (Mic-Dox), liposomal quercetin (Lipo-Qu), and micellar quercetin (Mic-Qu).

#### Phase1. Effect of particle size on distribution in the periablational rim after RF ablation

Studies were performed in two models, representing the tumor and the necessary surrounding normal tissue that must be ablated to achieve an adequate ablation margin [1]. First, 16 single subcutaneous R3230 breast adenocarcinoma tumors were implanted. Animals were randomized to receive RFA combined with color-labeled fluorescent beads of three sizes (20, 100, and 500 nm) given I5 min post-RF (6 animals×2 time points, n = 12) or IV fluorescent beads treatment alone (control tumors; n = 4). Next, the left liver lobes of 16 normal (tumor-free) animals were treated with RF ablation/IV fluorescent beads (20 nm and 100 nm, 15 min post-RFA) (6 animals×2 time points, n = 12) and control IV fluorescent beads alone (control livers; n = 4). Animals were sacrificed at 4 and 24 hr post-treatment, and tissues harvested for histopathologic and fluorescent microscopic analysis and quantification.

#### Phase 2. Effect of carrier (20 nm micelles vs. 100 nm liposomes) on combination therapy (RF ablation with doxorubicin or quercetin)

Seventy single subcutaneous R3230 tumors were divided into the following 7 treatment arms (n = 10 per group): RF alone, RF ablation with liposomal or micellar doxorubicin (both formulations: 1 mg in 0.5 ml, given 15 min post-RFA), and RF ablation with either liposomal or micellar quercetin (each formulation, 0.3 mg in 0.5 ml, given either 24 hr pre- or 15 min post-RFA based upon prior studies using these two time points for liposomal quercetin [14]). Doxorubicin (an HIF-1α inhibitor) and quercetin (an HSP70 inhibitor) were selected as both agents have known suppressive effects on hypoxia and RF ablation-induced heat stress responses, respectively [14], [22]. Animals were sacrificed and tumors harvested 24 hr post-RFA. Outcome measures included tumor coagulation and immunohistochemistry (IHC) for HIF-1α and HSP70 (including rim thickness and % cell positivity/high powered field [hpf]).

#### Phase 3. Effect of nanocarrier on tumor growth and survival after RF ablation

A total of 30 single subcutaneous R3230 tumors were used. Animals were allocated to the following 4 treatment arms: RF ablation with liposomal or micellar doxorubicin (both formulations: 1 mg in 0.5 ml, given 15 min post-RFA; n = 8 each arm), and RF ablation with either liposomal or micellar quercetin (each formulation, 0.3 mg in 0.5 ml) given 15 min post-RFA (n = 7–8 each arm). The administration time for RF/quercetin studies was selected based upon the results of Phase 2. Tumor growth was measured daily and animals were sacrificed at a pre-determined endpoint of 30 mm mean tumor diameter or 60 days survival post-ablation, whichever came first. Outcome measures included tumor growth curves and Kaplan Meier analysis of survival rates.

#### Phase 4. Effect of nanocarrier on intratumoral drug delivery and retention after RF ablation

Here, 64 paired subcutaneous R3230 tumors were implanted in 32 animals. Animals were allocated to the following treatment arms: RF ablation of one tumor followed by either liposomal doxorubicin or micellar doxorubicin (IV, 1 mg in 0.5 ml, given 15 min post-RFA). The second, paired non-ablated tumor served as in internal control that was exposed to either IV liposomal or micellar doxorubicin alone. Animals were sacrificed at 4 different time points (1–72 hr post-RFA), for a total of 64 tumors (n = 4 treatments×4 time points×4 per group). Ablated and unablated tumors and the left liver lobes were harvested. Outcome measures included gross and histopathologic evaluation for tumor coagulation, and fluorescent quantitative studies for intratumoral doxorubicin

### Animal Models

For all experiments and procedures, anesthesia was induced with 0.1 ml intraperitoneal (IP) injection of a mixture of ketamine (100 mg/ml, Ketaject; Phoenix Pharmaceutical, St. Joseph, MO) and xylazine (50 mg/ml, Bayer, Shawnee Mission, KS). To maintain adequate anesthesia, increments of 0.01 ml were used when necessary. Animals were sacrificed with an overdose of 0.3 ml of the same mixture.

Experiments were performed using two animal tissues. The first is a well-characterized established R3230 mammary adenocarcinoma cell line implanted in female Fisher 344 rats (150±20 g; 14–16 weeks old, Charles River, Wilmington, MA) [5], [23]. Tumor implantation, evaluation, and preparation techniques were performed as previously described [5]. Briefly, one tumor was implanted into each animal by slowly injecting 0.3–0.4 mL of tumor suspension into the chest mammary fat pad of each animal via an 18-gauge needle. 1.3–1.5 cm solid non-necrotic tumors were used (18–21 days after implantation), randomized to different treatment arms. For Phase 3, the second (remote) tumor was generated using similar implantation technique, with injection into the abdominal subcutaneous space 3.5– 4 cm distal to the primary site of injection in the chest. The second model was normal liver in Fischer F344 female rats. After anesthesia induction, hair was removed at the incision site and the skin was cleansed with a disinfectant (70% EtOH). A 15 mm incision was made in a subcostal location to expose the left lobe of the liver. After completion of the procedure, the abdomen was closed in layers using 4–5 interrupted sutures. All intravenous injections were administered via IV tail injection, under complete anesthesia.

### RF Application

Conventional monopolar RFA was applied by using a 500-kHz RFA generator (model 3E; Radionics, Burlington, Mass), as has been previously described [5]. Briefly, the 1-cm tip of a 21-gauge electrically insulated electrode (SMK electrode; Radionics) was inserted into the center of the tumor or left liver lobe. RF energy was applied for 5 min with generator output titrated to maintain a designated tip temperature (70±2°C, continuous monitoring via a thermocouple in the electrode tip). This standardized method of RF application has been previously demonstrated to provide reproducible volumes of coagulation with use of this conventional RFA system [5], [23]. To complete the RF circuit, the animal was placed on a standardized metallic grounding pad (Radionics).

### Preparation and administration of adjuvant nanoagents

For fluorescent beads, three commercially available carboxylated fluorescent dyed-polystyrene microspheres were used (FluoSpheres; Invitrogen, Eugene, OR), representing three different sizes/colors: 20 nm/crimson (wavelengths, excitation 625 nm/emission 645 nm), 100 nm/orange (540/560), and 500 nm/yellow-green (505/515), which were best observed using the purple, red, and green microscope color filters, respectively (per the manufacturer). Prior to initiating our experiments, single-colored beads were used as positive controls and examined under all fluorescent filters to ensure absence of any fluorescence bleed-through artifacts.

For liposomal doxorubicin, a commercially available preparation (Doxil; ALZA Pharmaceuticals, Palo Alto, CA) was used. Quercetin-loaded liposomes were prepared such that liposome formulation was identical to Doxil, and as has been described [14].

Doxorubicin-loaded micelles were prepared as described [24]. Briefly, first PEG2000-DSPE micelles were prepared using a lipid film hydration method. The lipid film was formed from a chloroform solution of PEG2000-DSPE by removal of the organic solvent by rotary evaporation followed by freeze-drying. The film was hydrated with 10 mM PBS pH 7.4 at room temperature and mixed using a vortex device for 5 min to give a final lipid concentration of 40 mg/ml. The PEG2000-DSPE micelles were then mixed with equal volume of drug solution (4 mg/ml) and incubated at room temperature for 1 h. Free doxorubicin was separated from the doxorubicin-micelle solutions using Amicon centrifuge filters (MWCO = 30 kDa). The micellar doxorubicin concentration was 2 mg/ml after diluting the micelles with methanol using a Labsystems Multiskan MCC/340 microplate reader (Labsystems and Life Sciences International, UK) at excitation and emission wavelengths of 485 and 590 nm, respectively. The micelle size (hydrodynamic diameter) was measured by dynamic light scattering (DLS) using a N4 Plus Submicron Particle System (Coulter Corporation, Miami, FL, USA) and was found to be 17.0±2.1 nm. The zeta-potential was −21.7±4.3 mV.

Quercetin-loaded micelles were also prepared using a lipid film hydration method. Briefly, 0.6 mg of quercetin (1 mg/mL solution in methanol) was added to polyethylene-glycolphosphatidyl-ethanolamine (PEG2000-PE) solutions in chloroform, and a lipid film was formed in a round-bottomed flask by solvent removal on a rotary evaporator. The lipid film was then rehydrated with 1 mL of phosphate buffered saline (pH 7.4) to obtain final lipid concentration 5 mM. After mixing using a vortex device for 15 min at room temperature, the unincorporated quercetin was removed by filtration of the micelle suspension through 0.2 µm membrane filters. The micellar loading efficiency of quercetin was 100% (as for quercetin-loaded liposomes, 0.6 mg of quercetin was loaded in each administered dose). The micelle size was 17 nm±2.1 nm. The zeta-potential was -21.7±4.3 mV.

### Tumor specimen retrieval

For Phases 1–3, tumors were removed from the animal and sectioned perpendicularly to the direction of electrode insertion. Tissue samples were split and processed for gross pathologic assessment of tumor coagulation, and for histopathology, immunohistochemistry, fluorescent microscopy, or doxorubicin quantification, as below.

### Confocal microscopy and fluorescent quantification

Tumor sections were flash frozen in optimal cutting temperature (OCT) media, to allow analysis of fluorescence. Tissues were sectioned at a thickness of 5 µm. For each tumor one slide was stained for H&E for gross pathology comparison and slides prepared for confocal fluorescent microscopy were counterstained with DAPI nuclear staining. A Zeiss LSM 510 Inverted Live-Cell Confocal System (Carl Zeiss Microscopy, Thornwood, NY) was used for image acquisition and tiling. In brief, slides were counterstained with Gold anti-fade reagent with DAPI (Life Technologies, Grand Islands, NY) and stored overnight, followed the next day with image acquisition. For each sample, at 10× and 40× magnification, a minimum of 100 fields were imaged and automatically tiled by the microscope software, Ziess LSM image examiner (Carl Zeiss Microscopy). Tiled images allowed subjective assessment and quantification of slices of the tumor section that encompassed the center, periablational rim and tumor edge. Images were then quantified for fluorescence using Volocity 6.0 software (PerkinElmer, Waltham, MA). For each tumor section, the peak values, means and sums of each fluorescent color surface area count were quantified. Where “peak values” represent the area with highest uptake, typically the periablational rim (as confirmed by duplicate H&E slides), “means” represent the average fluorescent surface area count per HPF and the “sums” represent the area under the curve (AUC) or total fluorescent surface area of a certain color in an entire section.

### Gross pathologic evaluation

To assess gross tumor coagulation, one half of each sample was incubated in 50 ml of PBS with 1 mg of 2,3,5-triphenyltetrazolium chloride (TTC, Sigma Aldrich) as has been previously described [5]. Non-viable white tissue, representing the coagulation zone, was identified and measured using manual calibers and recorded.

### Immunohistochemical staining

Tumor samples were placed in cassettes containing the central section of tumor. All tissues were fixed in 10% formalin overnight at 4°C, embedded in paraffin, and sectioned at a thickness of 5 µm. Tissues were stained with H&E for gross pathology. For Phase 2, at least 3 samples from each treatment group underwent immunohistochemical staining using previously described techniques [13], [14], [21]. Staining was performed using antibodies to HSP70, (Stressgen, Chicago, MI) [25], to detect evidence of HSP production, and HIF-1α (Abcam, Boston, MA) were used to detect the α-subunit of HIF-1.

Specimen slides were imaged at the periablational rim at 10× and 40× magnification and analyzed using a Micromaster I microscope (Fisher Scientific, Pittsburgh, PA) and Micron Imaging Software (Westover Scientific, Inc., Mill Greek, WA) to determine rim thickness and percent (%) cell positivity. Five random high power fields were analyzed for a minimum of 3 specimens for each parameter and scored in a blinded fashion to remove observer bias. As an additional control to insure uniformity of staining, whenever direct comparisons were made, immunohistochemical staining was repeated with all relevant comparison slides stained at the same time. Accuracy of the final data was verified by the senior author, who was blinded to treatment group.

### Drug quantification in harvested tissues

Doxorubicin inherent fluorescent properties were used to quantify drug accumulation in tumor and liver samples, as described [5]. In Phase 3, tissue was harvested from the ablated tumor, and remote, untreated tumor and untreated liver as controls. Tissues were weighed, and homogenized in acid alcohol (0.3N HCL, 70% EtOH), and doxorubicin was extracted for 24 hr at 4°C. Doxorubicin extracted from tissue homogenate supernatant samples was quantified by fluorometry with an excitation wavelength of 470 nm and intensity of emission measured at 590 nm and plotted on a standard curve.

### Statistical Analysis

The Microsoft Office 2010 Excel software (Microsoft, Redmond, WA) was used for statistical analysis. All data were provided as mean plus or minus SD. Immunohistochemistry results and fluorescence quantification were compared using analysis of variance (ANOVA). Additional post-hoc analysis was performed with paired, two-tailed Student's T-test, if and only if, the analysis of variance achieved statistical significance. A *P* value of less than 0.05 was considered significant. The Kaplan–Meier method and log-rank test were used for endpoint survival analysis. Given the absence of censoring of our data, one-way analysis of variance was then performed on the survival endpoints for each animal for the comparisons reported. Pair-wise t tests (p<0.05; two-tailed test) based on the least square means were subsequently performed only if the overall *P* values were significant.

## Results

### Phase 1. Smaller beads (20 nm) have greater deposition and deeper interstitial penetration than larger beads in the post-RFA periablational zone

**Figure 1:**
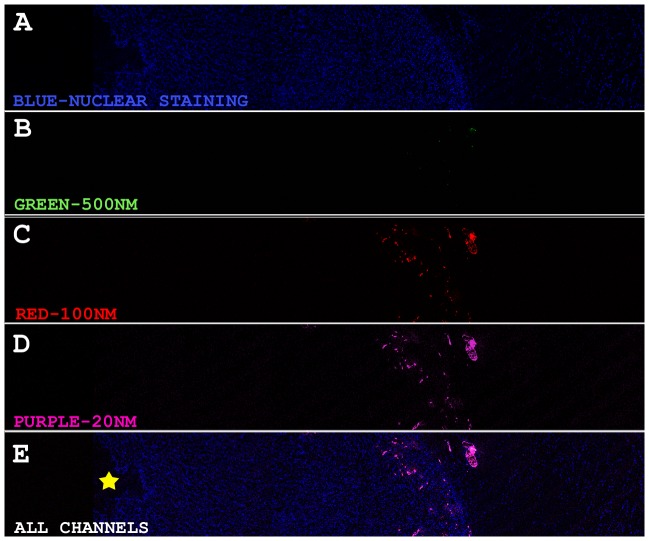
Confocal tiled Imaging for fluorescent surface area quantitation in R3230 tumors sacrificed at 4 hours post RF (10×). R3230 tumors were treated with RF alone, followed by IV injection of equal volumes of 3 fluorescent beads of different colors and sizes (purple 20 nm, red 100 nm, green 500 nm). Quantitation of tiled images of tumor sections (center, periablational rim and tumor margin) demonstrated fluorescent bead accumulation in the periablational rim, with greatest uptake of 20 nm beads (**D**) followed by the 100 nm (**C**) beads followed by the 500 nm beads (**B**) (p<0.05, all comparisons).

In R3230 tumors treated with RFA and IV fluorescent beads in animals sacrificed at 4 and 24 hr, greatest deposition was observed in the periablational rim, with peak uptake at 4 hr post-RFA for all sizes of beads. At 4 hr post-RFA, 20 nm beads had the greatest peak, sum and mean of fluorescence deposition, as depicted by fluorescent surface area detected followed by 100 nm beads, with the least uptake seen for 500 nm beads (**TABLE 1**). Similarly at 24 hr post-RFA, 20 nm beads had the greatest deposition as compared to 100 nm and 500 nm beads (p<0.05 for all comparisons, **TABLE 1** **,** **FIGURE 1** **,** **2**). Similarly, for normal liver we observed greater deposition of smaller beads (20 nm) in the periablational margin compared to larger beads after RF ablation. However, unlike the tumor model, bead deposition peaked later at 24 hr post-RFA (**TABLE 1**).

**Figure 2:**
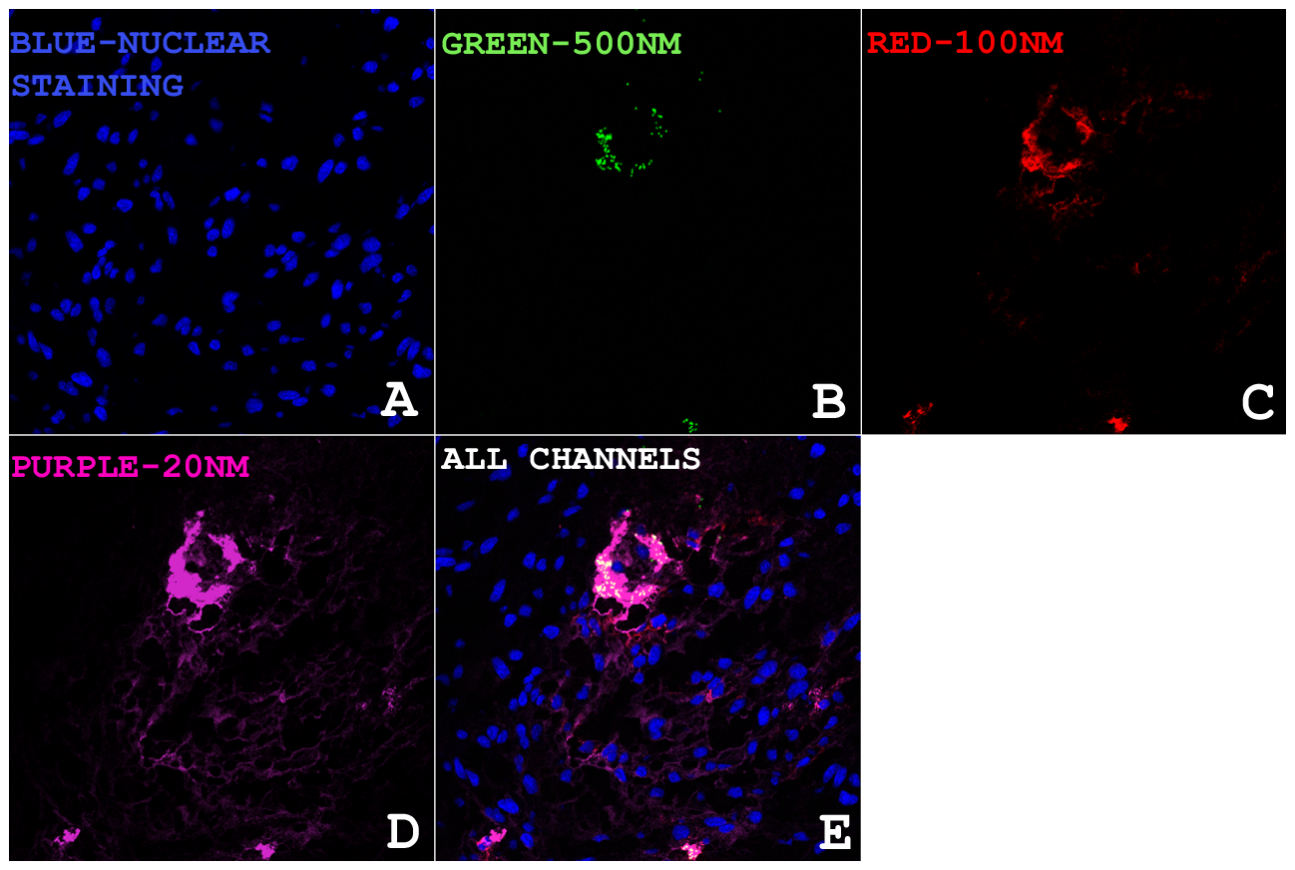
Confocal Imaging of perivascular and interstitial fluorescent bead penetration in the periablational rim 24 hr after RF ablation of R3230 tumors (40×). R3230 tumors were treated with RF alone, followed by IV injection of 3 fluorescent beads of different colors and sizes (purple 20 nm, red 100 nm, green 500 nm). 40× images of the periablational rim reveal deeper penetration of the 20 nm beads into the intracellular spaces beyond the primary site of extravasation, outlining and mapping out the cells they are surrounding (**D,E**), whereas the majority of the 100 nm (**B**) remain confined to the primary site of extravasation. Even less extravasation is seen for the 500 nm beads (**C**).

**Table 1:** Fluorescence for different sized beads in the periablational rim after RF of R3230 tumors.

Fluorescent Area ± STDV (µm^2^) at 4 hr after RF ablation of R3230 tumor								
	4 hrs post RF				24 hrs post RF			
	20 nm	100 nm	500 nm	P value	20 nm	100 nm	500 nm	P value
**Peak uptake**	13922±3281	5412±3116	947±347	*,**	5707±1205	2881±1205	1019±46	*,**,***
**Total uptake (AUC)**	15699±2178	6649±2670	1560±692	*,**,***	15835±3859	5215±182	2930±344	*,**,***
**Mean uptake (per HPF)**	1445±494	613±254	139±42	**,***	1288±90	783±243	330±71	*,**,***

* p<0.05 when comparing 20 nm values with 100 nm values per single time point.

**p<0.05 when comparing 20 nm values with 500 nm values per single time point.

***p<0.05 when comparing 100 nm values with 500 nm values per single time point.

### Phase 2. Adjuvant micellar Dox/Qu led to greater suppression of stress and hypoxia markers compared to adjuvant liposomal Dox/Qu preparations

**Figure 3:**
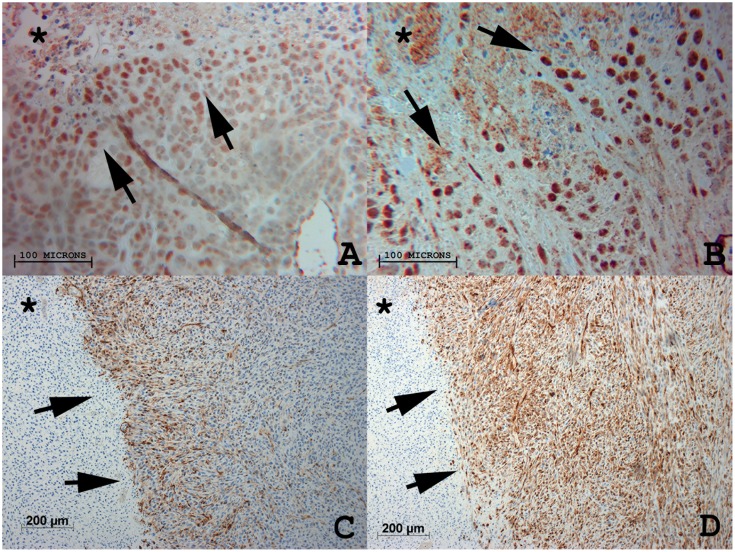
Comparison of micellar and liposomal formulations on modulating local periablational target proteins (HIF-1α and HSP70) 24 hr after RF ablation of R3230 tumor. (**A**) Micellar doxorubicin suppressed periablational HIF-1α expression to a greater degree than (**B**) liposomal doxorubicin 24 hr after RF ablation (40×). Similarly, (**C**) micellar quercetin suppressed ablation-induced periablational HSP70 expression in R3230 tumor at 24 hr compared to (**D**) liposomal quercetin (10×).

RFA combined with Mic-Dox led to greater suppression of HIF-1α expression compared to RFA combined with Lipo-Dox as measured by rim thickness in the periablational rim (45±37 µm vs. 129±68 µm, respectively, p<0.04), with equal % cell positivity (14.2±1.1 vs.13.6±2.1, respectively, p = 0.7) (**TABLE 2** **,** **FIGURE 3**). Both adjuvant Mic-Dox and Lipo-Dox therapies resulted in statistically significant reduction of HIF-1α expression as compared to RFA alone groups (210±85 µm and 37.9±4.3% at 24 hr, p<0.05 for all comparisons; **TABLE 2** **,** **FIGURE 3**).

**Table 2:** Gross and histopathologic outcomes for RFA/nanodrug combinations.

	Coagulation zone (mm)		Marker	Rim thickness (µm)		% Cell Positivity	
	Mean ± SD	P value		Mean ± SD	P value	Mean ± SD	P value
**RF alone**	7.7±0.6		HSP70	3000±236		80.2±5.6	
			HIF-1α	210±85		43.2±8.2	
**RF+Lipo Dox**	10.5±0.9	*	HIF-1α	129±68		14.2±3.3	*
**RF+Mic Dox**	11.3±2.3	*	HIF-1α	45±37	*,**	15.7±4.3	*
**RF+LipoQu 15 min post RF**	12.4±1.7	*	HSP70	2089±569	*	43.3±7.7	*
**RF+MicQu 15 min post RF**	11.4±1.5	*	HSP70	859.42±262	*,**	42.8±6.4	*
**RF+LipoQu 24 hr pre RF**	13.7±2.0	*	HSP70	1488±326	*	38.9±9.4	*
**RF+MicQu 24 hr pre RF**	13.1±0.9	*	HSP70	853±157	*,**	33.1±11.1	*

*  = p<0.05 when compared to RF group.

** = P<0.05 when compared to adjuvant liposomal drug preparations.

**Figure 4:**
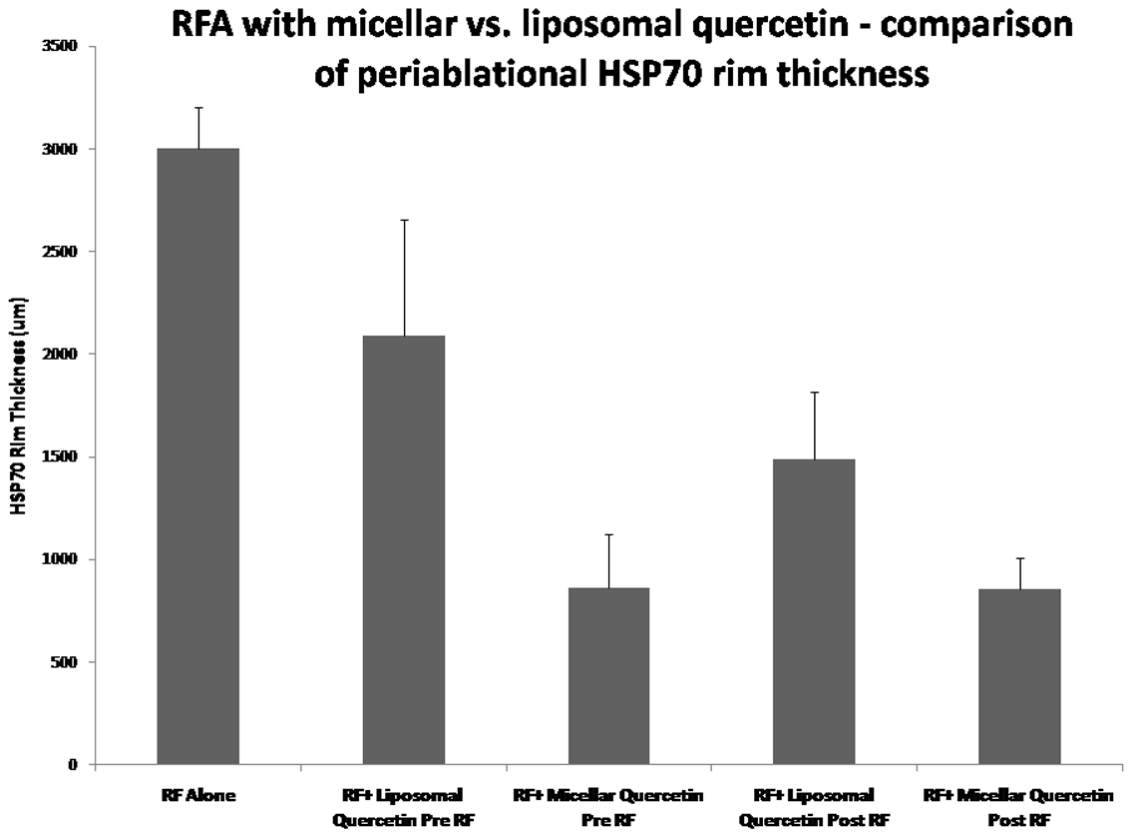
RF ablation combined with micellar quercetin suppresses periablational HSP70 expression more than a liposomal nanocarrier. Interestingly, in addition to the observed superior inhibitory effect of adjuvant micellar quercetin over adjuvant liposomal quercetin, regardless of timing of admininstration (pre-RFA or post-RFA), micellar adjuvant therapy is equally effective when given pre- or post-RFA (853.07±156.59 µm and 859.42±261.51 µm). However, adjuvant liposomal quercetin shows significantly greater HSP70 inhibition when given pre-RFA as compared to tumors treated with liposomal quercetin post-RFA (14888.01±325.53 µm and 2088.58±568.54 µm).

RFA combined with any quercetin nanopreparation administered at any time point reduced periablational HSP70 expression compared to RFA alone control groups (p<0.01 for all comparisons; **TABLE 2**). However, RFA micellar quercetin preparations markedly reduced rim thickness of HSP70 expression compared to RFA combined with liposomal quercetin for both 24 hr pre-RFA and 15 min post-RFA administration (853±157 µm vs. 14888±326 µm and 859±262 µm vs. 2089±569 µm, respectively, p<0.03 for all comparisons) (**FIGURE 4**). With regard to timing of adjuvant nanodrug administration, Mic-Qu markedly reduced rim thickness of HSP70 expression equally at both 24 hr pre- and 15 min post-RFA administrations (853±157 µm and 859±262 µm, respectively, p = NS). However, adjuvant Lipo-Qu resulted in the greatest suppression of RFA-induced HSP70 expression when given 24 hr pre-RFA, with significantly less effect on the rim thickness of HSP70 expression when given 15 min post-RFA (14888±326 µm and 2089±569 µm, respectively; p<0.01) (**TABLE 2**).

### Phase 3. RF ablation combined with long-circulating liposomal nanodrugs led to equal local tumor coagulation and equal or better control of tumor growth and animal endpoint survival compared to micellar nanodrugs

Significantly greater tumor coagulation was achieved in treatment groups combining RFA with any adjuvant nanopreparations of quercetin or doxorubicin, than by RFA alone at 24 hr (all comparisons p<0.05, **TABLE 2**). Yet, no statistically significant difference in coagulation was observed based on type of drug or nanopreparation used at 24 hr (**TABLE 2**).

**Figure 5:**
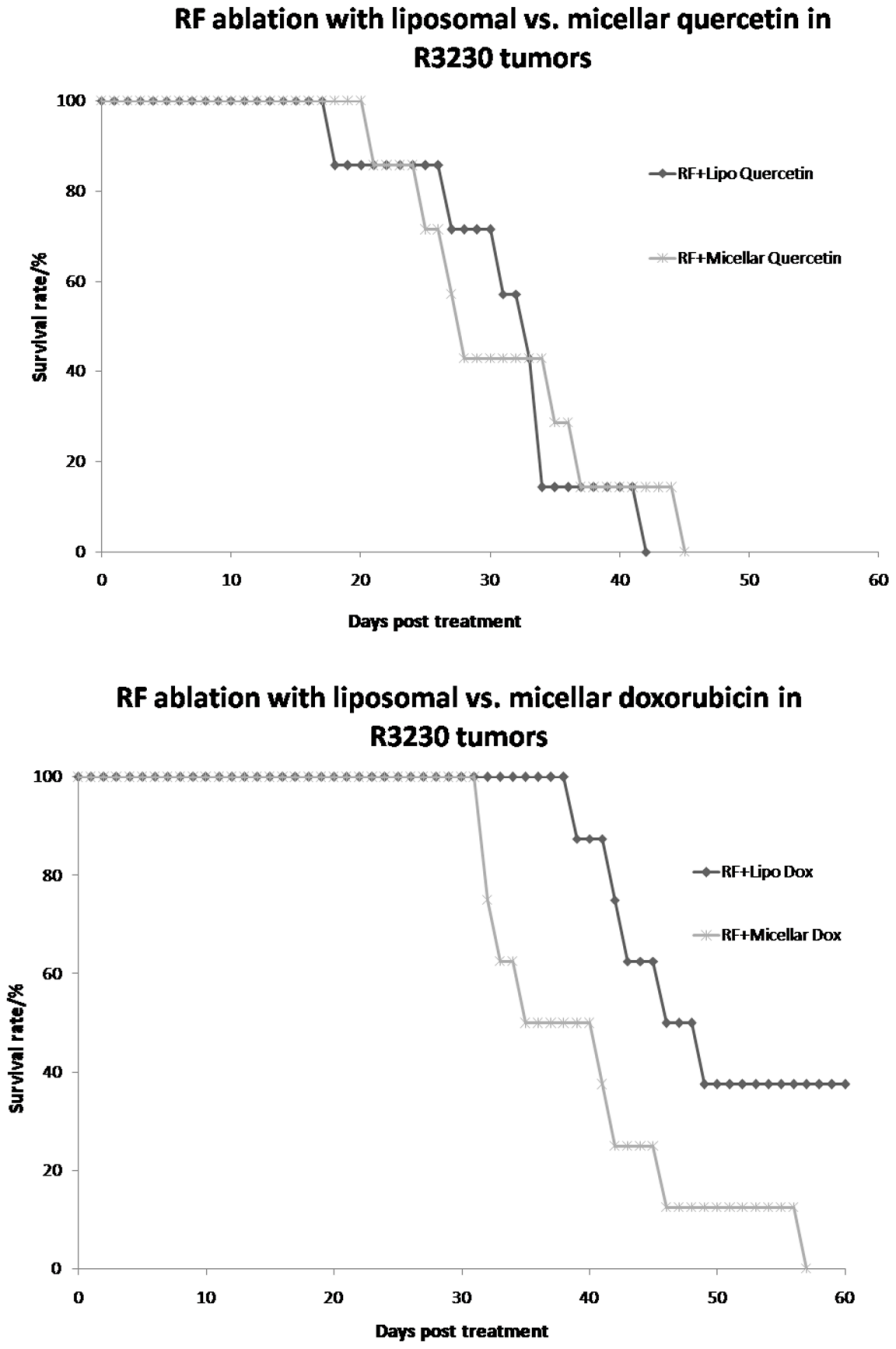
Comparison of animal endpoint survival for micellar and liposomal doxorubicin or quercetin formulations when combined with RF ablation of R3230 tumors. (**A**) RF/Lipo-Dox resulted in the greatest animal survival (49.8±9.1 d), followed by RFA/Mic-Dox (39.6±8.4 d). (**B**) RFA combined with either micellar quercetin or liposomal quercetin resulted in the same mean animal survival (31.1±8.2 d and 31.2±9.1 d, respectively).

For RFA combined with doxorubicin nanopreparations, the mean endpoint survival for animals treated with RFA/Lipo-Dox was 49.8±9.1 d, including two animals that survived up to the 60 day post-treatment monitoring end-point. This was significantly longer than RFA/Mic-Dox, which had a mean survival of 39.6±8.4 d (p<0.04, **FIGURE 5**). By contrast, RFA/Lipo-Qu and RFA/Mic-Qu had similar animal endpoint survival profiles, where RFA/Mic-Qu had a mean survival of 31.1±8.2 d compared to 31.2±9.1 d with RFA/Lipo-Qu (p = 0.9, **FIGURE 5**).

### Phase 4. Contrasting intratumoral doxorubicin accumulation kinetics for micellar and liposomal preparations after RF ablation

At 1 hr post-RFA, there was minimal doxorubicin detected in tumor samples of either Mic-Dox or Lipo-Dox groups. However, at 4 hr post-RFA, intratumoral doxorubicin levels were approximately 6-fold higher for RFA/Mic-Dox compared to RFA/Lipo-Dox (0.0006±0.0002 µg/g vs. 0.0001±0.000024 µg/g, p<0.03). Yet, at the later time points of 24 hr and 72 hr, RFA/Lipo-Dox increased to a much greater degree than micellar doxorubicin concentrations. This resulted in significantly higher levels of intratumoral doxorubicin (approximately 17-fold) for long-circulating liposomal doxorubicin delivery compared to RFA/Mic-Dox (p<0.01 for all comparisons, **TABLE 3**). Specifically, at 24 hr post-RFA, doxorubicin levels in the periablational rim were 1.02±0.52 µg/g for RFA/Lipo-Dox compared to only 0.06±0.02 µg/g for RFA/Mic-Dox (p<0.001). Similarly, at 72 hr post-RFA, intratumoral doxorubicin levels after RFA/Lipo-Dox were 1.17±0.52 µg/g, compared to RFA/Mic-Dox 0.01±0.02 µg/g (p<0.001).

**Table 3:** Periablational intratumoral doxorubicin accumulation for liposomal and micellar nanocarriers combined with RF ablation over time.

Doxorubicin accumulation in RF ablated tumors (µg/g)		
Time post RF	RF+Lipo Dox	RF+Mic Dox
	Mean ± SD	Mean ± SD
1 hour	0.00000081±0.0000003	0.00000007±0.0000001
4 hours	0.0001±0.000024	0.0006±0.000174*
24 hours	1.02±0.517*	0.06±0.024
72 hours	1.17±0.243*	0.01±0.013

*  = p<0.05 when compared to the other treatment group.

## Discussion

There is increasing interest in developing treatment paradigms that combine focal tumor ablation with adjuvant pharmacologic or chemotherapeutic agents to address both challenges with residual viable tumor from incomplete local treatment and difficulties in achieving high concentrations of targeted drug delivery [18], [26]–[28]. Early studies combining RF ablation with a commercially-available liposomal doxorubicin (Doxil) preparation reported increases in local tumor coagulation, periablational drug uptake, and reduced tumor growth in animal studies, and increased tumor destruction in preliminary clinical studies [5], [6], [8], [9]. Subsequent studies have refined the approach, either through modification of the drug payload or using thermosensitive preparations to facilitate intratumoral drug release, though with mixed improvements in treatment efficacy [11]–[13], [17]. While most of these studies have largely used 100 nm sized liposomes as the carrier model, more recent studies on nanoparticle delivery (without ablation) suggest improved carrier and drug penetration with smaller-sized preparations [20], [29].

In our current study, smaller-sized particles (20 nm) administered in combination with RF ablation did indeed result in greater and deeper interstitial and perivascular penetration into the periablational rim compared to larger (100 nm and 500 nm) sized particles. Thus, our findings are consistent with uses of smaller-sized carriers to overcome limitations in intratumoral and interstitial drug delivery when using nanoparticles alone or in combination with low-level hyperthermia (40–45°C) applications [29], [30]. For example, Tsukioke et al have reported deeper interstitial penetration of micellar doxorubicin into tumor spheroids as compared to liposomal doxorubicin [29]. Yet, our results go well beyond these findings as we achieved a primary goal of demonstrating that the greater nanodrug penetration from micellar preparations can be translated into markedly improved modulation/suppression of specifically targeted tissue reactions in the periablational rim. To wit, here ablation-induced expression of both HIF-1α and HSP70 were independently suppressed to a greater degree with micellar nanopreparations compared to their liposomal counterparts in terms of spatial distribution. Specifically, micellar nanodrugs specifically reduced the geographic extent of marker expression (i.e., rim thickness) in the periablational rim, highlighting greater tissue penetration micellar preparations with concurrent greater spatial distribution of the desired biochemical modulation.

While these results are exciting in achieving a primary goal of using refined nanodrug paradigms to target specific cellular reactions, it must be acknowledged that these gains in interstitial penetration and suppression of local tissue reactions do not necessarily translate into improvements in all desired outcome metrics in cancer therapy. Along these lines, in our study micellar preparations had similar or inferior effects on curbing tumor growth and promoting animal survival and likewise micellar preparations did not increase local ablation-induced tumor coagulation compared to liposomal preparations at 24 hr. This reinforces our understanding that local effects of tumor coagulation and periablational proteomic reactions may also not directly translate into reduction in tumor growth and gains in animal endpoint survival outcomes.

To account for the results we observed, we hypothesize that marked, but incomplete reduction of HSP or HIF-1a may be insufficient to induce complete tumor destruction at 24 hr and that, the ability of each drug to increase local coagulation may be susceptible to a threshold effect, with only a certain amount of drug required to target the partially injured remaining cells in the periablational rim. However, other longer-term effects on growth in the remaining untreated tumor may reflect a more complex reality where differences in nanocarrier release and drug uptake that are greater with long-circulating liposomal formulations [31] may very well be of primary importance for overall survival. Indeed, our results are concordant with previously reported findings by Yang et al, where RFA combined with liposomal paclitaxel (an apoptosis enhancer) resulted in greater apoptosis (as measured by caspase 3), yet did not suppress tumor growth to a similar extent as liposomal doxorubicin [13]. Conversely, with increasing evidence that some reactions (such as increased HIF-1α) in the periablational rim may stimulate growth in distant tumor, successful modulation of local tissue reactions may assume increasing clinical relevance as its own endpoint separate from tumor growth [32]–[34].

Our study adds to the growing body of evidence supporting the notion that it is the variable nanodrug delivery kinetics that are likely to be primarily responsible for the way that different nanopreparations induce better or worse effects on specific outcome metrics [17], [35]. For example, while greater intratumoral doxorubicin uptake was observed with micelles compared to liposomes early (4 hr) after RF ablation, liposomes delivered significantly greater doxorubicin to the treated tumor over a longer period of time (24–72 hr post-treatment). Therefore, while micellar preparations had a greater effect on our specific targets (HIF-1α and HSP70), these markers also peak early (4–24 hr post-RFA) and may be more susceptible to early drug delivery. In contrast, if the overall amount of drug delivery to the tumor is the primary goal, then long-circulating liposomal preparations are superior carriers. Greater overall tumor exposure to accumulating doxorubicin may explain the greater animal endpoint survival observed with RFA/liposomal doxorubicin. Along these lines, recently, Andriyanov et al reported corroborating findings when comparing RF ablation combined with conventional long-circulating stealth PEG-ylated liposomal doxorubicin (i.e., Doxil) to fast-releasing 100 nm thermosensitive liposomal doxorubicin (i.e., ThermoDox), in which prolonged slow drug uptake resulted in a greater reduction in long-term tumor growth compared to early flooding of the tumor with intratumoral nanodrug [17]. Indeed, we posit that longer drug circulation, and therefore exposure time, may take on greater importance for processes that are likely to occur over a variable relatively longer time frame in the target tumor. Specifically, doxorubicin, an intercalating agent, is more effective when cells are in the G2 phase of replication, and cells in and around the ablation zone are likely to enter the G2 phase at variable time points after ablation [36]). Having high concentrations of doxorubicin in the serum when various cells enter this point of the cell cycle over a period of days may well represent the best chance to ensure that efficacious drug concentrations are present when most needed.

Additional potentially useful pharmacologic observations were noted in our study. For quercetin nanopreparations, we also found a wider window of efficacious administration with micellar preparations than may have been expected given the well-known, relatively short (2–3 hr half-life) plasma kinetics for these smaller vehicles [37]. Specifically, we demonstrated equivalent responses and tumor coagulation when micellar quercetin was given between 24 hr pre- to 15 min post-RF ablation compared to the liposomal formulation where optimal HSP70 suppression was observed only when given 24 hr pre-RFA. Thus, our results suggest that intratumoral concentrations are primary over plasma kinetics and that likely very little drug is needed to achieve the desired HSP reduction – provided it is successfully delivered to the desired spatial location. Additionally, we observed variable timing of accumulation for smaller fluorescent bead particles between tumor and normal liver, where peak fluorescence was observed at 24 hr post-RFA in normal liver. This suggests that even with equivalent outcomes, some types of nanocarriers, with ideal therapeutic windows tailored to specific tissues, may offer clinically relevant and practical advantages.

Ultimately, our findings suggest that several different factors need to be considered when developing combination therapy paradigms. Optimal choice of carrier type and drug payload will likely involve both identifying goals of treatment and prioritizing outcome metrics (e.g., local suppression of a specific cytokine such as HIF-1α or control of tumor growth). Tumor and tissue-specific characteristics, and differences in carrier and drug pharmacokinetics will factor into the practical considerations of when to time peri-ablation drug administration. Thus, the choice of nanoparticles should ultimately likely be tailored to achieve specific goals in specific tissues, as optimal carriers may differ depending on whether modulation of local periablational processes or growth suppression of untreated tumor is required.

We acknowledge several limitations with our study. Fluorescent beads were commercially acquired and the number of beads per volume was pre-determined by the manufacturer and was not likely not identical between different particle sizes. Yet, we controlled for the key variable drug concentration in all of our subsequent experiments as the active drug (i.e., 1 mg of doxorubicin loaded in 0.5 ml) was given precedence to the number of particles in the micellar or liposomal vehicle. Thus, as expected, fluorescent beads of a smaller size had a consistently higher concentration than larger-sized beads. Additionally, although the R3230 model used for these studies is a well-characterized tumor model, results demonstrated may be specific to the model and should be interpreted and applied to other scenarios and models with caution, mirroring our call for tailoring nanopreparations to different tumor types and scenarios. Differences also exist in pharmacokinetic profiles between the fluorescent beads and correspondingly sized nanopreparations (i.e., 20 nm beads vs. 20 nm micelles). Therefore, interpretation and correlation of results between fluorescent beads and nanopreparations must be made carefully. Furthermore, in survival studies, we did not include an RFA alone treatment arm, as prior studies have clearly demonstrated a survival benefit for combination therapy arms (liposomal doxorubicin or quercetin) [9], [38]. Finally, tumor coagulation for micellar doxorubicin in this study differs from results obtained in an earlier study using a small liposome preparation (∼40 nm), where high levels of intratumoral doxorubicin accumulation but smaller amounts of tumor coagulation were reported compared to a 100 nm preparation. Differences in results may represent variable micellar stability due to differences in doxorubicin loading doses [29], and emphasizes that results from experimental and clinical studies using combination paradigms are critically dependent on developing the appropriate nanopreparations for the right application.

In conclusion, when combined with RF ablation, smaller-sized particles have superior deeper tissue penetration and therefore can potentially achieve more effective local molecular modulation of specific post-ablation reactions including heat shock protein and HIF-1α expression, with a wider window of administration as compared to larger (100 nm) particles. However, larger-sized long-circulating particles can result in greater overall long-term intratumoral drug accumulation and reduced tumor growth. Therefore, different nanodrug carriers provide specific advantages, in part based upon size and circulation kinetics, which should be considered when formulating strategies to achieve optimal combination therapies with tumor ablation.
